# Anaerobic degradation of a mixture of MtBE, EtBE, TBA, and benzene under different redox conditions

**DOI:** 10.1007/s00253-018-8853-4

**Published:** 2018-02-24

**Authors:** Marcelle J. van der Waals, Charles Pijls, Anja J. C. Sinke, Alette A. M. Langenhoff, Hauke Smidt, Jan Gerritse

**Affiliations:** 1 Deltares, Subsurface and Groundwater Systems, Daltonlaan 600, 3584 BK Utrecht, the Netherlands; 2 Laboratory of Microbiology, Wageningen University & Research, Stippeneng 4, 6708 WE Wageningen, the Netherlands; 3 Tauw, Handelskade 37, 7400 AC Deventer, the Netherlands; 4 BP International Limited, Sunbury on Thames, Middlesex, TW167BP UK; 5 Department of Environmental Technology, Wageningen University & Research, Bornse Weilanden 9, 6708 WG Wageningen, the Netherlands

**Keywords:** MtBE, EtBE, TBA, Benzene, Anaerobic degradation, Electron acceptors, Cometabolism

## Abstract

The increasing use of biobased fuels and fuel additives can potentially change the typical fuel-related contamination in soil and groundwater. Anaerobic biotransformation of the biofuel additive ethyl *tert*-butyl ether (EtBE), as well as of methyl *tert*-butyl ether (MtBE), benzene, and *tert*-butyl alcohol (TBA, a possible oxygenate metabolite), was studied at an industrially contaminated site and in the laboratory. Analysis of groundwater samples indicated that in the field MtBE was degraded, yielding TBA as major product. In batch microcosms, MtBE was degraded under different conditions: unamended control, with medium without added electron acceptors, or with ferrihydrite or sulfate (with or without medium) as electron acceptor, respectively. Degradation of EtBE was not observed under any of these conditions tested. TBA was partially depleted in parallel with MtBE. Results of microcosm experiments with MtBE substrate analogues, i.e., syringate, vanillate, or ferulate, were in line with the hypothesis that the observed TBA degradation is a cometabolic process. Microcosms with ferulate, syringate, isopropanol, or diethyl ether showed EtBE depletion up to 86.5% of the initial concentration after 83 days. Benzene was degraded in the unamended controls, with medium without added electron acceptors and with ferrihydrite, sulfate, or chlorate as electron acceptor, respectively. In the presence of nitrate, benzene was only degraded after addition of an anaerobic benzene-degrading community. Nitrate and chlorate hindered MtBE, EtBE, and TBA degradation.

**Electronic supplementary material:**

The online version of this article (10.1007/s00253-018-8853-4) contains supplementary material, which is available to authorized users.

## Introduction

The use of renewable resources, such as bioethanol, as basis for automotive fuels is stimulated under the European Biofuels Directive (EC 2018) to reduce greenhouse gas emissions. Both fossil-based and biobased fuels may enter the groundwater through incidents and spillages. An overview compiled by Concawe (division of the European Petroleum Refiners Association) showed that oxygenates are present in groundwater, drinking water, surface water, runoff water, precipitation, and air in the European environment (Stupp et al. 2012). The oxygenate methyl *tert*-butyl ether (MtBE) is a synthetic volatile organic compound added to gasoline to increase its octane number, i.e., performance. Due to its high water solubility, low odor threshold, and health concerns, MtBE contamination is a widespread problem that requires remediation (Deeb et al. 2003; van Wezel et al. 2009). Although the tertiary carbon structure and stable ether bond suggest that the MtBE molecule will not be readily degraded by microorganisms under anaerobic conditions, degradation has been observed under a variety of redox conditions, including methanogenic, nitrate-reducing, manganese-reducing, iron-reducing, and sulfate-reducing conditions (Bradley et al. 2001a; Finneran and Lovley 2001; Fischer et al. 2005; Häggblom et al. 2007; Hyman 2013; Liu et al. 2016; Mormile et al. 1994; Pruden et al. 2005; Somsamak et al. 2005; Somsamak et al. 2006; Suflita and Mormile 1993; Waul et al. 2009; Wilson et al. 2005). Key metabolites of aerobic MtBE degradation are *tert*-butyl alcohol (TBA), *tert*-butyl formate (TBF) and 2-hydroxy isobutyric acid (HIBA) (Fiorenza and Rifai 2003). The detection of TBA indicates that ether bond cleavage is the initial step in anaerobic MtBE degradation. It has been found that under anoxic conditions TBA is often a recalcitrant product of MtBE degradation (Schmidt et al. 2004). However, mineralization of MtBE without accumulation of TBA has been shown under denitrifying and oxic conditions (Bradley et al. 2001b).

In the past years, MtBE has been partially replaced by ethyl *tert*-butyl ether (EtBE), a biofuel oxygenate synthesized from (bio)ethanol and isobutylene. Due to its high solubility and persistence, EtBE disperses rapidly in the environment (Le Digabel et al. 2013). Little is known about anaerobic EtBE degradation. Several studies have reported lack of any anaerobic degradation of EtBE (Hernandez-Perez et al. 2001; Mormile et al. 1994; Somsamak et al. 2001). In contrast, Yeh and Novak demonstrated EtBE degradation in soils under denitrifying and methanogenic conditions (Yeh and Novak 1994). Compound-specific stable isotope analysis (CSIA) revealed insignificant carbon isotope fractionation, and low hydrogen isotope fractionation up to + 14‰ along an anoxic EtBE plume at a contaminated field site in the Central West of Spain suggesting anaerobic biodegradation of EtBE (Bombach et al. 2015).

In addition to TBA being produced as metabolite during MtBE and EtBE degradation, it is also frequently used as a polar organic solvent in flavors, perfumes, or paint removers. TBA is highly soluble in water and is considered as a widespread contamination problem (Deeb et al. 2003). Several studies in the absence or presence of MtBE have concluded that TBA is either degraded with rates ranging between <0.14 and 2 μM/day/g dry soil or persists in anoxic groundwater (Bradley et al. 1999; Mormile et al. 1994; Somsamak et al. 2001; Yeh and Novak 1994). Under methanogenic conditions no TBA degradation has been observed (Bradley et al. 2002; Mormile et al. 1994). TBA consumption in anoxic microcosms with iron(III) as electron acceptor has been demonstrated in microcosms (Finneran and Lovley 2001). A direct link between TBA oxidation and electron acceptor reduction has, however, not been demonstrated. Aerobically, TBA can be degraded via 2-methyl-2-hydroxy-1-propanol, HIBA, 2-propanol, acetone, and hydroxyacetone to carbon dioxide and water.

Benzene is a natural constituent of crude oil, has a high octane number, and is commonly present in (bio)fuel blends (Chin and Batterman 2012). Massive production and use of benzene combined with its high mobility, relatively low anaerobic biodegradation rates, and carcinogenicity make benzene one of the most widespread groundwater contaminants of concern. Therefore, benzene is typically considered as risk determining compound in fuels. Anaerobic benzene biodegradation has been described under denitrifying, iron-reducing, sulfate-reducing, and methanogenic conditions (Coates et al. 2002).

Several studies have investigated the effects of mixtures of fossil-based and biobased fuels on degradation of the single components. Both antagonistic and synergistic effects have been reported during degradation of mixtures of benzene, toluene, ethylbenzene, xylene (BTEX), and MtBE. The presence of BTEX was shown to enhance aerobic MtBE degradation by a mixed microbial culture under continuous flow conditions (Sedran et al. 2002). On the contrary, biodegradation of MtBE in the subsurface was inhibited largely by the presence of ethylbenzenes and xylenes and partially inhibited by benzene and toluene until MtBE had migrated beyond the BTEX plume (Deeb et al. 2001). The preferential utilization of ethanol under aerobic, denitrifying, iron-reducing, sulfate-reducing, and methanogenic conditions resulted in a negative effect on BTEX degradation (Corseuil et al. 1998; Lovanh et al. 2002; Ruiz-Aguilar et al. 2002). Bioaugmentation with a benzene-enriched methanogenic consortium enhanced anaerobic benzene degradation in a mixture of BTEX and ethanol (Da Silva and Alvarez 2004).

The concurrence of MtBE, EtBE, TBA, and benzene may affect the microbial degradation potential for both fossil-based and biobased components. Nevertheless, to our knowledge, studies on the anaerobic degradation of a mixture of MtBE, EtBE, TBA, and benzene have not been reported. In this study, anoxic groundwater from a contaminated location was used to determine the microbial degradation potential for a mixture of benzene, MtBE, EtBE, and TBA, the latter being a degradation product of MtBE and EtBE.

The aims of the present study were therefore to (i) determine the potential of an indigenous microbial community from a contaminated site for the anaerobic degradation of MtBE, EtBE, TBA, and benzene in a mixture; (ii) determine the effect of electron acceptors nitrate, sulfate, amorphous 2-line ferrihydrite, and chlorate on the anaerobic degradation; and (iii) determine anaerobic TBA degradation and stimulate this process using MtBE and EtBE substrate analogues and potential metabolites as substrates.

## Materials and methods

### Field site, modeling, and sampling procedures

Experiments were focused on MtBE, EtBE, TBA, and benzene contaminated groundwater at an industrial site. At this site, the redox conditions varied from iron reducing to methanogenic, and the pH was around 7 (Table S1). At the impacted area, the contamination was located between 9 and 20 m depth below the surface in an anoxic aquifer. Two source zones of MtBE were present with a maximum MtBE concentration of about 4000 μM. The highest concentrations of EtBE, TBA, and benzene detected in 2009 were 1 μM (88 μg/l), 130 μM (9636 μg/l), and 260 μM (20,309 μg/l), respectively. The average MtBE, TBA, and benzene concentrations per layer in 2009, 2013, and 2015 are given in Table S2. The data for MtBE, TBA, and benzene depletion at the field site were compared with an existing hydrological model and contaminant fate and transport model that was calibrated using field data on measured heads, porosity, organic matter content, and long-term diver data on groundwater fluctuations (Harbaugh 2005; Harbaugh et al. 2017).

Groundwater was collected from a monitoring well located in one of the MtBE source zones, in gas tight bottles of 1 litre. The bottles were filled completely with groundwater to prevent the introduction of oxygen, were transported in a cool box with ice the same day to the laboratory, and were stored at 4 °C for preparation of microcosms within 14 days after sampling.

### Microcosm incubations

Microcosms were prepared under a 80/20% N_2_/CO_2_ (*v*/*v*) flow in 250-ml serum bottles (Glasgerätebau Ochs GmbH, Bovenden, Germany) and crimp sealed with viton rubber stoppers (Rubber BV, Hilversum, The Netherlands) and aluminum crimp caps (Grace, MD, USA). Traces of O_2_ were stripped from the N_2_/CO_2_ gas as described previously (van der Waals et al. 2017). The microcosms contained 90 ml of groundwater contaminated with MtBE, TBA, and benzene and amended with 50 μM EtBE added from a 20 mM anoxic, autoclaved stock solution. One series of microcosms with different electron acceptors was amended with 10 ml of 10-fold concentrated anoxic, sterile mineral medium with vitamins that was prepared as described previously, but without the addition of nitrate (van der Waals et al. 2017). No medium was added to a second series of microcosms with the addition of only electron acceptors. Electron acceptors, i.e., nitrate (NaNO_3_), chlorate (NaClO_3_), iron (amorphous 2-line ferrihydrite) (Schwertmann and Cornell 2007), or sulfate (Na_2_SO_4_), were added from separately autoclaved stock solutions of 70.5 mM to final concentrations of 2.4 mM. The pH of the prepared medium was 7.0. The microcosms were incubated upside down and continuously shaken at 100 rpm (Certomat, B|Braun, Melsungen, Germany) in the dark at 20 °C. Abiotic control microcosms were autoclaved and contained 100 mg/l HgCl_2_ and 2 mg/l NaN_3_. Unamended control microcosms, not receiving any medium and/or electron acceptors, were used as representatives for the natural condition. When MtBE or benzene was depleted, the compound was added to approximately 100 μM using diluted anoxic, autoclaved 20 mM stock solutions.

### TBA cometabolism experiment

Serum vials (10 ml, Grace) with an 80/20% N_2_/CO_2_ (*v*/*v*) headspace, crimp sealed with a viton rubber stopper and aluminum crimp cap were prepared with medium and 5 mM methanol (Merck, Darmstadt, Germany), isopropanol (Sigma-Aldrich, MO, USA), diethyl ether (Sigma-Aldrich), syringic acid (Fluka analytical, St. Louis, USA), ferulic acid (Sigma-Aldrich), or vanillic acid (Boom, Meppel, the Netherlands) neutralized with 1 M NaOH stock solution, respectively. These substrates were selected, since they are potential metabolites of MtBE and TBA degradation (methanol and isopropanol, respectively), or because they may be EtBE and MtBE analogues containing ethoxy (diethyl ether) or methoxy groups (syringic acid, ferulic acid, and vanillic acid), respectively. Serum vials were filled with 2.4 ml liquid from an unamended control or medium microcosm, respectively. The TBA concentration in the vials was regularly measured using solid phase microextraction (SPME) on a gas chromatograph with flame ionization detector (GC-FID) as described below. Concentrations of methanol, ethanol, isopropanol, and diethyl ether in the serum vials were also regularly measured using a GC-FID equipped with an SPME fiber.

TBA (Sigma-Aldrich), MtBE (Rathburn Chemicals, Walkerburn, Scotland), benzene (Janssen Chimica, Beerse, Belgium), and EtBE (Sigma-Aldrich) were of a purity of > 99%.

### Analytical procedures

Headspace samples of 0.5 ml were taken from the microcosms to measure methane using a 1-ml sterile syringe (B|Braun) and 0.5 × 25 mm needle (Henke Sass Wolf, Tuttlingen, Germany) and measured on a Varian 3800 GC-FID as previously described (van der Waals et al. 2017). Liquid samples of 0.3 ml for measuring MtBE, EtBE, TBA, and benzene were taken from the microcosms using a 1-ml sterile syringe (B|Braun) and 0.5 × 16 mm needle (Henke Sass Wolf) and injected in a 10-ml headspace vial (Grace) containing 7 ml of 20 mg/l mercury chloride and 2 ml of 0.93 μg/ml 1-propanol as internal standard (Acros, Geel, Belgium). SPME was performed on these aqueous solutions using a 75-μm CAR/PDMS 24ga autosampler fiber, which was conditioned according to the manufacturer’s protocol (Sigma-Aldrich). Six standards from 10 to 200 μM in serum bottles crimp sealed with viton stoppers and aluminum caps were used for calibration. An extraction time of 30 min and a desorption time of 3 min were found as optimum (Fig. S1). A Combi-PAL autosampler (CTC analytics, Zwingen, Switzerland) with SPME fiber holder and agitator was used for this GC method. Each measurement sequence contained a calibration line and quality controls (Milli-Q and calibration sample) within the sequence because of degrading fiber adsorption potential due to high benzene concentrations (Black and Fine 2001). The coefficient of variation of this method was 5%. The potential metabolites detected with this method include methanol, TBF, ethanol, TBA, acetone, isopropanol, and *N*-propanol.

Ion analyses were done using 0.1 ml supernatant of shortly (5 s) centrifuged samples (5000×*g*) (Eppendorf centrifuge 5424, Hamburg, Germany) diluted up to 1 ml with Milli-Q water and measured on a Dionex ICS-1500 equipped with an Ionpac AS19 anion-exchange column and an AERS 500 suppressor (Dionex Corp., Sunnyvale, CA). The potassium hydroxide gradient to separate anions was as follows: 10 mM from 0 to 10 min and 10 to 45 mM from 10 to 25 min with a flow rate of 1 ml/min. The sample injection volume was 25 μl.

Compound stable isotope analysis (CSIA) was performed in 2009 (Supplementary information) and 2013 by Hydroisotop (www.hydroisotop.de) using isotope ratio mass spectrometry (GC-IRMS) related to the Vienna PDB standard. Groundwater samples were fixed in the field with a pellet sodium hydroxide in 100-ml serum bottles.

### DNA extraction and molecular analyses

Biomass was concentrated from 10 ml microcosm samples by vacuum filtration on 0.2 μm pore-size filters (Merck Millipore). Filters were crushed with a sterilized wooden tooth pick and total DNA was extracted using the MoBio Powerlyzer DNA isolation kit (MoBio, CA, USA). DNA was stored at − 80 °C until further molecular analyses. The total bacterial 16S rRNA gene and the *abcA* gene encoding benzene carboxylase were quantified as described previously (van der Waals et al. 2017). For this study, a quantitative real-time PCR assay was designed using the Primer3 software (Koressaar and Remm 2007; Untergasser et al. 2012) to detect genes coding for isobutyryl-CoA mutase (*icmA*) that has been shown to be a key enzyme involved in MtBE degradation in *Aquincola tertiaricarbonis* (Rohwerder et al. 2006). The designed primer pair (F695) 5′-ACATCTCGGGCTACCACATC-3′ (R868) 5′-CCTCGAAGAAGTCACCTTGC-3′ with TaqMan probe 5′-CTGGCCAACCTGATCACCTACGT-3′ (757) was evaluated in silico using the publicly accessible NCBI BLAST search tool (Ye et al. 2012). The *icmA* gene assay was performed in 25 μl volumes as described elsewhere (van der Waals et al. 2017). The temperature program on an IQ5 Icycler (Bio-rad, Veenendaal, the Netherlands) was 5 min at 95 °C followed by 45 cycles of 20 s at 95 °C, 60 s at 58 °C and a final elongation step of 3 min at 72 °C. Normalized *abcA* and *icmA* gene counts were calculated as a concentration relative to the bacterial 16S rRNA gene count.

### First-order degradation rate constant calculation

First-order degradation rate constants (*k*) were calculated according to Eq. 1 (Suarez and Rifai 1999).

1 $$ k=\left(\frac{\log \left({x}_1\right)-\log \left({x}_2\right)}{t_2-{t}_1}\right) $$

where *t*_2_ − *t*_1_ is the time interval in days; *x*_1_ is the concentration at time *t*_1_ and *x*_2_ is the concentration at time *t*_2_.

## Results

### Field data

In 2015, MtBE concentrations had decreased strongly compared to measurements in 2009 and 2013. Based on the average concentration in all measured monitoring wells, only 2% of the MtBE was left in 2015 (Table S2). Benzene concentrations had decreased in the shallow layers of the aquifer up to 25 m below surface in 2015 as compared with 2013. MtBE, TBA, and benzene concentrations did not change in the deeper layer of 25–30 m below surface from 2009 till 2015. In 2009, the δ^13^C for MtBE ranged from − 24 to − 31‰. In 2013, MtBE δ^13^C had increased up to + 50‰. The TBA δ^13^C was constant ranging from − 27 to − 31‰ over 2009–2013. Overall, the fate and transport model achieved a fairly good correlation with long-term benzene and MtBE field monitoring data at a normalized root mean square deviation (NRMSD) of about 14%. Although the limited dataset of TBA demonstrated a NRMSD of about 14%, the overall observation was that correlation of TBA field data was unsatisfactory. In order to fit the TBA plume, complete lack of degradation was assumed. First-order degradation rate constants for benzene and MtBE of 0.0007 day^−1^ in the model were representative for the field plume situation.

In field samples from 2010, an average 16S rRNA total bacterial gene count of 5.0 ± 8.5 × 10^5^ gene copies/ml sample was detected. The functional gene, *icmA*, was detected in 3 out of 14 groundwater monitoring wells. Normalized gene counts were relatively low, ranging from 1.5 × 10^−5^ to 2.7 × 10^−4^. The functional gene, *abcA*, was detected in 2009, 2013, and 2015 at stable concentrations with an average concentration of 1.2 ± 2.4 × 10^−4^ relative to the total bacterial 16S rRNA gene copies.

### Biodegradation of MtBE, EtBE, TBA, and benzene in microcosms

In microcosms with groundwater, MtBE was degraded in the unamended control and with the addition of medium and ferrihydrite, at first-order rate constants of 0.02 ± 0.002 day^−1^, 0.07 day^−1^, and 0.04 day^−1^, respectively (Table 1). Under sulfate-reducing conditions with the addition of medium, the MtBE first-order degradation rate constant of 0.06 day^−1^ was higher than without the addition of medium (0.03 day^−1^) (Table 1). The concentration data for benzene, MtBE, and electron acceptors were used to calculate electron balances. Under ferrihydrite-reducing conditions, more electrons were released from benzene and MtBE (assuming complete oxidation to CO_2_) than the electrons used to reduce iron(III) to iron(II) with no detectable net production of metabolites (Table S3). Under sulfate-reducing conditions, fewer electrons were released from benzene and MtBE than the electrons used to reduce sulfate to sulfide (Table S3). In sterile control microcosms no degradation was observed.

**Table 1:** First-order degradation rate constants (*k*) ± standard deviation over multiple spikes in the microcosms under different redox conditions

Condition	MtBE [day^−1^]		MtBE after re-addition^a^ [day^−1^]	Benzene [day^−1^]		Benzene after re-addition^a^ [day^−1^]
	− medium	+ medium		− medium	+ medium	
Unamended control	0.02 ± 0.002		0.02 ± 0.005	0.01 ± 0.005		–
Medium addition without added electron acceptors		0.07	0.03 ± 0.03		0.02	–
Nitrate addition	ND	ND	ND	ND	0.02^b^	0.08 ± 0.04^b^
Chlorate addition	ND	ND	ND	0.01	0.39	–
Ferrihydrite addition	0.04	0.02	0.03 ± 0.003	0.01	0.08	–
Sulfate addition	0.03	0.06	0.02	0.02	0.07	–

*ND* no degradation up to 940 days, − no benzene re-added to the microcosm

^a^With medium addition, except for the unamended control

^b^Degradation rate constant with a 5% (*v*/*v*) inoculation of culture liquid from a benzene-degrading, denitrifying biofilm community

EtBE did not show any degradation after 1140 days in the microcosms under different redox conditions, even after depletion of MtBE and benzene.

**Fig. 1:**
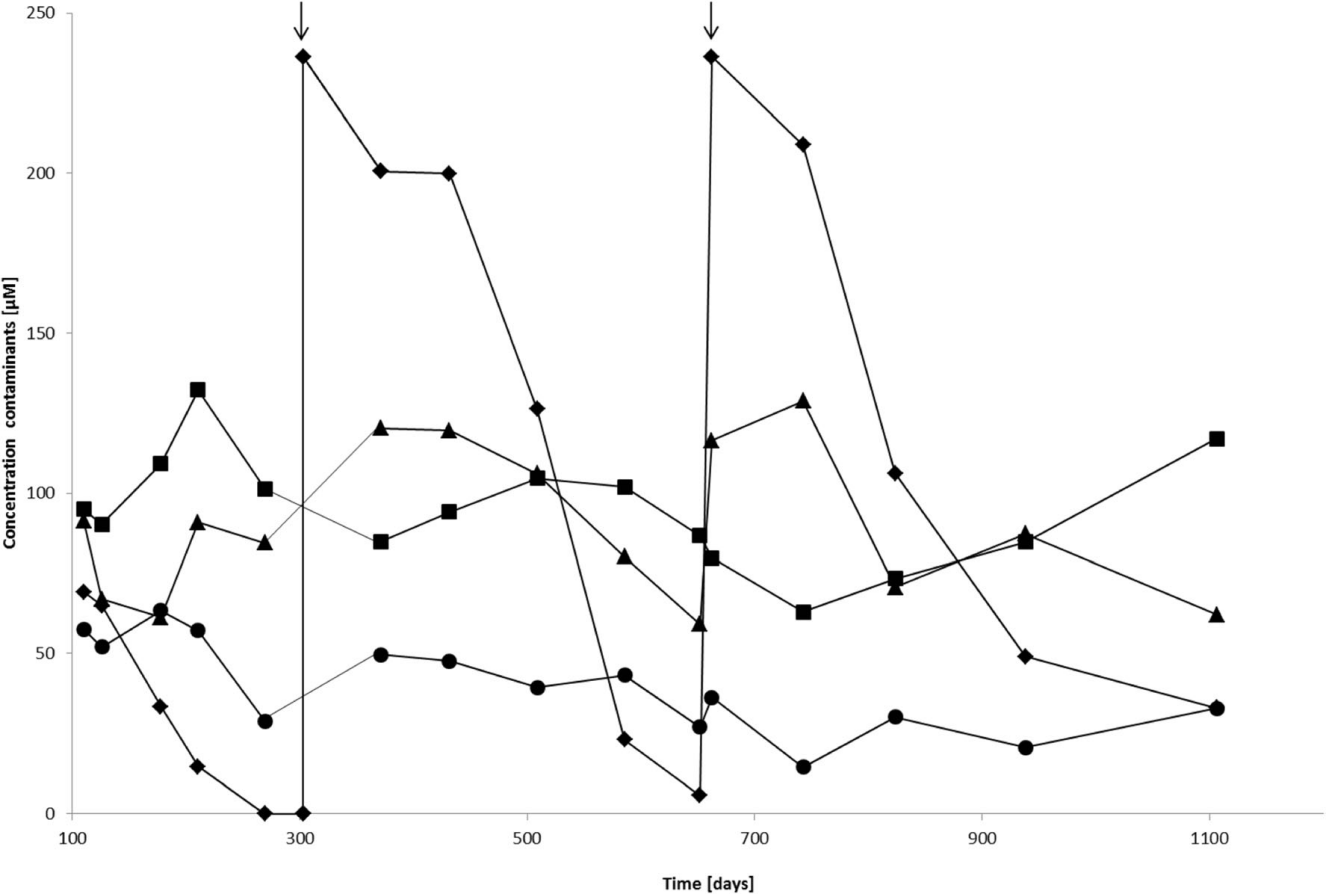
Concentration profile under natural conditions of a mixture of MtBE (diamonds), EtBE (squares), TBA (triangles), and benzene (circles). The arrows indicate a re-addition or MtBE

MtBE was re-added to the different microcosms after depletion, and an increase in TBA concentration was observed in medium without added electron acceptors, ferrihydrite-reducing or unamended microcosms corresponding to a percentage of 8, 30, and 19 ± 5%, respectively, compared to the concentration of MtBE degraded. The MtBE and TBA concentration profile under natural conditions is shown in Fig. 1. This TBA increase leveled out to the initial TBA concentration after about 200 days. Complete TBA mineralization has not been proven. After a second MtBE dosage, the TBA concentration increase corresponded to 1%, 34%, and 24 ± 7% of the MtBE degraded in medium, ferrihydrite-reducing and unamended microcosms, respectively. The TBA increase was leveled out for this second dosage after 80 days.

Benzene was degraded in unamended controls and with the addition of medium without added electron acceptors, chlorate, ferrihydrite, or sulfate, with first-order degradation rate constants between 0.01 and 0.02 day^−1^ (Table 1). In the presence of medium, the benzene first-order degradation rate constants were significantly higher than in the absence of medium under chlorate (0.39 day^−1^), ferrihydrite (0.08 day^−1^), or sulfate-reducing conditions (0.07 day^−1^) (Table 1). No degradation with nitrate was observed. The electron balance indicated that more electrons were used to reduce chlorate to chloride than were released by the oxidation of benzene (Table S3). Initially, no benzene degradation was observed in the nitrate-reducing microcosms. Supply of a 5% liquid inoculum from a benzene-degrading, denitrifying biofilm culture grown on benzene and nitrate with medium resulted in an average degradation rate constant of 0.08 ± 0.04 day^−1^ (van der Waals et al. 2017). Over two dosages of 100 μM benzene, on average 5.6 ± 3.1 mol nitrate was reduced in parallel with degradation of 1 mol benzene. No increased methane concentration was measured.

Total bacterial 16S rRNA genes in the microcosms were on average 2.0 ± 1.3 × 10^6^ gene copies/ml sample. The *icmA* gene was only detected in the microcosms under nitrate-reducing conditions at normalized counts of 6.7 × 10^−5^ and 1.6 × 10^−5^ in the absence or presence of medium, respectively. The *abcA* gene was detected in 14 out of 20 microcosms at a normalized count of 5.0 ± 5.2 × 10^−3^. In the microcosms with addition of medium and the microcosm with groundwater plus nitrate, *abcA* genes were below the detection limit (< 1.1 copies/μl sample or < 6.0 × 10^−4^ relative to the total bacterial 16S rRNA gene copies). The *abcA* gene copy number in the microcosm with medium plus nitrate and reactor inoculum was 5.8 times higher than the bacterial 16S rRNA gene number.

### Anaerobic TBA and EtBE degradation during growth on MtBE and EtBE analogues and potential metabolites

**Fig. 2:**
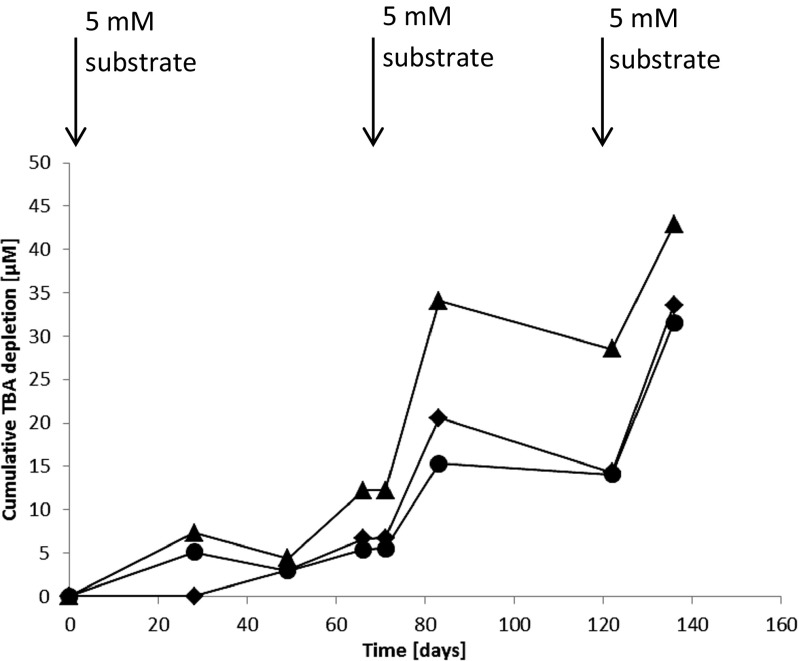
The cumulative TBA depletion with vanillate (triangles), ferulate (diamonds), or syringate (circles) in MtBE-degrading groundwater microcosms with medium. The arrows indicate a 5-mM dosage of the substrate vanillate, ferulate, or syringate, respectively. An initial TBA concentration of 50 μM was present in the microcosms

Microcosms with vanillate, ferulate, syringate, isopropanol, methanol, or diethyl ether as growth substrate analogues were prepared to determine whether TBA was cometabolically degraded. TBA was depleted with all substrates tested (Table 2). After two re-additions of 5 mM syringate, ferulate, or vanillate, to microcosms with medium, the cumulative amount of TBA depleted increased to 31.6, 33.6, and 42.9 μM, respectively in 136 days (Fig. 2). The amount of vanillate, ferulate, or syringate added was, respectively, 350, 447, or 475 mol/mol of TBA depleted. Also EtBE depletion was observed at significant amounts in microcosms with ferulate (both unamended control and medium), syringate (unamended control and medium), isopropanol (unamended control), or diethyl ether (unamended control and medium), respectively (Table 2).

**Table 2:** EtBE and TBA depletion [%] after 83 days in microcosms with samples from the unamended control or medium condition, respectively, plus 5 mM analogue substrate. Initially the MtBE, EtBE, and TBA concentration were 50 μM. At 71 days, 5 mM vanillate, ferulate, or syringate was re-added to the specific medium microcosms

Analogue substrate	EtBE depletion unamended control / medium [%]	TBA depletion unamended control / medium [%]
Vanillate	0 / 0	26.3 / 62.5
Ferulate	86.5 / 33.9	93.8 / 45.7
Syringate	36.4 / 37.6	25.2 / 35.1
Isopropanol	15.7 / 0	18.2 / 22.3
Methanol	0 / 0	18.7 / 45.8
Diethyl ether	55.8 / 44.7	19.6 / 30.6

## Discussion

The aims of this study were to (i) determine the biodegradation potential of the microbial community for MtBE, EtBE, TBA, and benzene in a mixture in groundwater from a contaminated location; (ii) determine the effects of electron acceptors; and (iii) determine TBA and EtBE degradation using MtBE and EtBE substrate analogues and potential metabolites. A better understanding of the degradation rates under different redox conditions and cometabolic TBA depletion would enable the design of more effective bioremediation through biostimulation at contaminated sites.

### Anaerobic MtBE degradation

MtBE concentrations decreased strongly in all monitoring wells from 2009 to 2015. The detection of TBA in almost all monitoring wells in 2013 indicates that demethylation is the first step in the MtBE biodegradation in the aquifer (Youngster et al. 2010). Fractionation of stable carbon isotopes of MtBE and TBA in groundwater in 2009 and 2013 confirmed degradation of MtBE. TBA degradation could not be demonstrated by CSIA.

MtBE was anaerobically degraded in microcosms with groundwater in unamended controls and after the addition of medium, ferrihydrite, or sulfate. This indicates that conditions prevailing at the field site favor MtBE degradation in batch cultures. The MtBE degradation rate constant implemented in the model and representative for the field situation of 0.0007 day^−1^ was 25 times lower compared with the rate observed in the enrichments (0.02 ± 0.002 day^−1^). Although data obtained in batch are only indicative for the field situation, these outcomes suggest the potential of biostimulation and/or augmentation of laboratory enriched microbial communities to increase the MtBE degradation rate in the field. The first-order MtBE degradation rate constant of 0.02 ± 0.002 day^−1^ in the unamended control was similar compared with previous rate constants found of 0.01–0.09 day^−1^ in methanogenic microcosms (Liu et al. 2016; Somsamak et al. 2006; Wilson et al. 2005). The first-order MtBE degradation rate constants obtained in this study under sulfate-reducing conditions of 0.03 and 0.06 day^−1^ in the absence and presence of medium, respectively, are comparable with a previous microcosm rate constant of 0.031 ± 0.007 day^−1^ (Wilson et al. 2005). The TBA concentration did not stochiometrically increase with the amount of MtBE degraded in the microcosms with sulfate. This suggests that MtBE may be completely oxidized to CO_2_ or other metabolites were formed, which were not detected with the GC-FID. The electrons released from MtBE and benzene degradation were in the range of 60 to 83% compared with the electrons used to reduce sulfate to sulfide. Somsamak et al. (2006) and Youngster et al. (2008) indicated that in their experiments anaerobic MtBE degradation was not directly coupled to sulfidogenesis (Somsamak et al. 2006; Youngster et al. 2008). They hypothesize that MtBE-degrading organisms might have produced acetate which was used as a carbon source for sulfate-reducing organisms. Up to our knowledge, this study found the highest first-order MtBE degradation rate constant under ferrihydrite-reducing conditions of 0.04 day^−1^. Landmeyer et al. (1998) reported a first-order rate constant of 0.0002 day^−1^ under iron-reducing conditions in microcosms (Landmeyer et al. 1998). In the ferrihydrite microcosms, much more benzene and MtBE was consumed in comparison to the amount of ferrihydrite that was reduced. This indicates that ferrihydrite reduction may not be the predominant electron accepting reaction. The electron imbalance could also be attributed to a lack of complete oxidation of benzene and MtBE to CO_2_. The fact that there was no iron(II) production in the sterile controls suggests that abiotic ferrihydrite reduction did not compromise/influence the measurements.

We hypothesized that *icmA* gene products might also be relevant in anaerobic MtBE degradation since (i) cobalamin-dependent enzymes are important in the metabolism of many anaerobes and (ii) no oxygenases are needed for the transformation of 2-HIBA to 3-hydroxybutyryl-CoA and the further metabolism of this intermediate (Janssen and Schink 1993). In the field, low concentrations of *icmA* genes were detected in three out of 14 monitoring wells. In microcosms, *icmA* was only detected under nitrate-reducing conditions at low normalized counts of 6.7 × 10^−5^ and 1.6 × 10^−5^. This low *icmA* concentration suggests that 2-hydroxyisobutyrate is not, or, slowly degraded. It may also be that under anaerobic conditions other isobutyryl-CoA mutase enzymes were active, which were not detected by the applied *icmA* assay (Rohwerder et al. 2006), or that MtBE and TBA were degraded via alternative metabolic pathways, not involving isobutyryl-CoA mutase.

### Anaerobic EtBE degradation

No significant anaerobic degradation of EtBE was detected in the microcosms under different redox conditions, even after depletion of the (apparently) more easily degradable substrates MtBE and benzene. This result is in line with recent studies indicating the lack of anaerobic EtBE degradation (Hernandez-Perez et al. 2001; Somsamak et al. 2001). However, microcosms with ferulate, syringate, isopropanol, and diethyl ether demonstrated EtBE depletion of up to 86.5% of the initial concentration after 83 days. This suggests that bacteria capable of cometabolic EtBE degradation might be present at this location. We recommend additional research to confirm the possible cometabolic EtBE degradation during growth on methoxylated and ethoxylated substrates.

### Anaerobic cometabolic TBA depletion

Interestingly, TBA concentrations did not stoichiometrically increase in parallel with MtBE depletion in the field nor in the microcosm experiment. The initial TBA increase after the re-addition of MtBE and the subsequent TBA decrease during MtBE degradation to its initial concentration in the microcosms suggests cometabolic TBA degradation. It is feasible that TBA was not depleted further after the primary growth substrate was consumed. This is in line with previous studies observing simultaneous anaerobic MtBE and TBA degradation (Table 3). A strong TBA decrease in microcosms with vanillate, ferulate, or syringate indicates cometabolic degradation with MtBE analogue substrates. Possibly, TBA degradation was catalyzed by enzymes induced for demethoxylation, as was shown previously under aerobic conditions (Lopes Ferreira et al. 2006). It is also feasible that TBA degradation is mediated by enzymes responsible for metabolism of *tert*-alcohol intermediates, which are formed during degradation of methoxylated aromatics. Re-addition of substrate analogues to the medium microcosms doubled the amount of TBA depleted, indicating that these compounds indeed induced cometabolic TBA depletion. Without the addition of these substrates, TBA was not depleted below its initial concentration of 50 μM, and EtBE was not degraded at all. The amount of vanillate (350 mol), ferulate (447 mol), or syringate (475 mol) was added in excess to degrade 1 mol of TBA. Therefore, the substrate concentration used in this study is rather high compared with a previously reported MtBE cometabolic degradation study using 1.6 mol vanillate or 1.7 mol syringate to degrade 1 mol of MtBE (Youngster et al. 2008). Further laboratory experiments will be important to test whether lower substrate concentrations can be used to cometabolically degrade TBA. Addition of the analogue growth substrates to the batches may have enriched microorganisms with enzymes catalyzing MtBE, EtBE, and TBA transformation. A previous study has demonstrated cometabolic MtBE degradation using syringate, vanillate, and guaiacol (Youngster et al. 2008). It was suggested that acetogenic bacteria capable of degrading methoxylated aromatics may be responsible for this enhanced MtBE degradation (Youngster et al. 2008). Anaerobic TBA degradation has also been observed in anaerobic microcosms without the addition of MtBE (Finneran and Lovley 2001). However, these microcosms contained aquifer sediment heavily contaminated with MtBE, suggesting residual MtBE adsorbed to the sediment might have caused possible cometabolic TBA degradation.

**Table 3:** The amount of TBA formed versus MtBE degraded [mol%] in anaerobic microcosms

Enrichment source	Amendment	Amount of TBA formed versus MtBE degraded [average ± st. dev in mol%]	References
MtBE-degrading culture in batch	Medium	4.4 ± 4.8	This study
MtBE-degrading culture in batch	2.35 mM iron + medium	31.6 ± 2.9	This study
MtBE-degrading culture in batch	None	21.5 ± 4.0	This study
10% sediment underwater site Arthur Kill estuarine inlet	None	100	Liu et al. (2016)
Sediment wash water, bioreactor sludge, Port Hueneme	9 mM ferric chloride	± 100	Pruden et al. (2005)
Sediment underwater site Arthur Kill estuarine inlet	None	54.5	Somsamak et al. (2005)
Coronado cays, estuarine site	20 mM sodium sulfate None	77.5 ± 4.9 97.5 ± 9.2	Somsamak et al. (2006)
Sediment underwater site Arthur Kill estuarine inlet	20 mM sodium sulfate	100	Somsamak et al. (2001)
Bed sediment Charleston Bed sediment Pensacola	4.6 mM nitrate 7.3 mM nitrate	0 1	Bradley et al. (2001b)
Sediment from Cecil Field, Oasis, and Picatinny Arsenal^a^	4.1 mM nitrate 126.5 mM Manganese(IV) 102.9 mM iron(III) 11.5 mM sulfate None	0 5.3 ± 1.7 7 ± 2 4.3 ± 2.7 9 ± 1	Bradley et al. (2001a)
Slurry Ohio River	5 mM sodium sulfate	100	Mormile et al. (1994)

^a^Average amount of TBA degradation calculated over the three different locations for the specific amendment

### Anaerobic benzene degradation

The benzene degradation rate constant implemented in the model and representative for the field situation of 0.0007 day^−1^ was 14 to 557 times lower than the first-order rate constants measured in microcosms. The benzene first-order degradation rate constants in microcosms under chlorate, ferrihydrite, and sulfate-reducing conditions were higher in the presence of medium. This implies that organisms responsible for benzene degradation were stimulated by nutrients or vitamins supplied with the medium to increase the benzene degradation rate. Initially, benzene was not degraded in microcosms under nitrate-reducing conditions. After a 5% (*v*/*v*) inoculation of culture liquid from a benzene-degrading, denitrifying biofilm community a first-order benzene degradation rate constant of 0.08 ± 0.04 day^−1^ was obtained. This rate constant is lower compared with a rate constant of 0.52 ± 0.03 day^−1^ found in a previous microcosm study with this biofilm community containing a dense inoculation of biofilm material from the glass walls of the continuous culture (van der Zaan et al. 2012). The stoichiometry of anaerobic benzene degradation, 1 mol benzene consumed per 5.6 ± 3.1 mol nitrate reduced, was similar to that found previously (van der Zaan et al. 2012). The incomplete electron recovery of benzene degradation with chlorate or sulfate indicates that electrons released from the relatively high concentration of DOC of 32 mg/l were used to reduce these acceptors in our microcosms. No increased methane concentration was measured in these microcosms, indicating that methanogenesis was not involved in benzene degradation.

The *abcA* gene associated with benzene carboxylation was detected in all monitoring wells and microcosms, albeit in low numbers. The normalized *abcA* gene numbers in the microcosms under different redox conditions (5.0 ± 5.2 × 10^−3^) were higher than the gene numbers measured in the field (1.2 ± 2.4 × 10^−4^). This suggests that the organisms responsible for benzene degradation through carboxylation were enriched in the microcosms by the addition of nutrients and/or electron acceptors. Groundwater from a leachate-contaminated aquifer polluted with BTEX contained on average 2.4 × 10^−4^ normalized counts of the *bssA* gene that codes for benzylsuccinate synthase, an enzyme that is important in toluene degradation (Staats et al. 2011). This relative number of *bssA* genes is similar to the *abcA* gene count in this study. Our results suggest that bioaugmentation and biostimulation can be used to enhance the anaerobic benzene degradation rate at this location. These results are in line with a previous study indicating that benzene is degraded through an initial carboxylation, and the responsible organisms may be stimulated by the addition of nutrients and nitrate (van der Waals et al. 2017). Interestingly, the microcosm containing nitrate plus medium and an inoculum from a benzene-degrading, denitrifying biofilm culture showed an *abcA* gene count of 5.8 times higher than the bacterial 16S rRNA gene number. This high *abcA* gene count could be due to (i) multiple *abcA* gene copies in the DNA of the biofilm culture or (ii) the possibility of the *abcA* gene being mobile due to flanking transposon sequences that were observed on the same contig in a metagenome dataset derived from the chemostat biofilm (unpublished data).

### Mixture experiment

Addition of nitrate plus a benzene-degrading community and chlorate (with and without medium) stimulated benzene degradation, but hindered MtBE, EtBE, and TBA degradation. TBA degradation may be significantly decreased by the presence of benzene in aerobic microcosms (Sedran et al. 2002; Sedran et al. 2004), while other studies indicated no effect of benzene on MtBE and TBA degradation in microcosms (Pruden and Suidan 2004). No EtBE degradation was observed in the mixture without substrate analogues. This result is in line with previous studies indicating (i) that under both aerobic and anaerobic conditions, structurally related compounds can have a negative effect on the degradation rates (Deshpande et al. 1987) or (ii) that MtBE might be preferentially degraded (Kharoune et al. 2001). Gunasekaran and colleagues reported inhibition of EtBE degradation in the presence of benzene, toluene, and xylene (Gunasekaran et al. 2013). Somsamak and colleagues also reported a lack of EtBE degradation in a mixture of MtBE, EtBE, and *tert*-amyl ether (TAME) (Somsamak et al. 2001). In this study, a stoichiometric increase of TBA with the degradation of MtBE was observed under sulfate-reducing conditions.

Addition of nitrate with a 5% (*v*/*v*) benzene-degrading culture inoculation and chlorate to the microcosms stimulated benzene degradation. For MtBE, EtBE, and TBA, electron acceptors such as nitrate appeared to hinder the degradation. Growth substrate analogues stimulated degradation. Cometabolic TBA depletion with MtBE and different methoxylated substrate analogues was indicated in this study. The addition of methoxylated compounds such as syringate, vanillate, and ferulate may be a useful method to enhance anaerobic MtBE, EtBE, and TBA degradation in situ. Further field studies and dedicated pilot testing could help to elucidate the potential of cometabolic TBA, MtBE, and EtBE degradation. The increasing likelihood of encountering mixtures of fossil-based and biobased fuels as cocontaminants in groundwater may require novel or sequential bioremediation approaches. This study describes that biostimulation with substrate analogues, medium and/or nitrate, chlorate, ferrihydrite, and sulfate and bioaugmentation approaches can potentially accelerate the degradation of MtBE, EtBE, TBA, and benzene.

## Electronic supplementary material

ESM 1 (PDF 123 kb)
