# Supplementary material for: Anaerobic degradation of a mixture of MtBE, EtBE, TBA, and benzene under different redox conditions
Source: Appl Microbiol Biotechnol. 2018 Feb 24;102(7):3387–97. doi: 10.1007/s00253-018-8853-4 (PMC5852185; doi:10.1007/s00253-018-8853-4)
Supplement: Supplementary file 1 — (PDF 123 kb) [file 253_2018_8853_MOESM1_ESM.pdf]

Applied Microbiology and Biotechnology

Anaerobic degradation of MtBE, EtBE, TBA and benzene under different redox conditions

Marcelle J. van der Waals[#1,2], Charles Pijls[3], Anja J.C. Sinke[4], Alette A.M. Langenhoff[5], Hauke Smidt[2] and Jan Gerritse[1]

[1]Deltares, Subsurface and Groundwater Systems, Daltonlaan 600, 3584 BK Utrecht, the Netherlands

[2]Wageningen University & Research, Laboratory of Microbiology, Stippeneng 4, 6708 WE Wageningen, the Netherlands

[3]Tauw, Handelskade 37, 7400 AC Deventer, the Netherlands

[4]BP International Limited, Sunbury on Thames, Middlesex TW167BP, UK

[5]Wageningen University & Research, Department of Environmental Technology, Bornse Weilanden 9, 6708 WG, Wageningen, the Netherlands

# Corresponding author

Phone +31 646914584, Fax +31 88 3357856, E-mail: Marcelle.vanderwaals@deltares.nl

Carbon Stable Isotope Analysis method 2009

For stable isotope analysis of MTBE and TBA, extraction from the water phase was carried out using the Stir Bar Sorptive Extraction (SBSE) technique (David and Sandra 2007). The stir bar was loaded with compounds, where after the compounds were thermally extracted from the stir bar in a thermal desorption unit 275°C (TDU, Gerstel, Germany) and introduced into the GC using a Cold Injection System injector (CIS, Gerstel, Germany). This allows measurement of stable isotope ratios of compounds at low concentration. The gas chromatograph (Agilent 6890 series) was equipped with an AT-1 column (30m x 0.32µm x 5µm). The GC temperature program was held at 50°C for 5 min, increased to 120°C at a rate of 10°C/min, isothermal at 5 min, followed by an increase to 300°C at a rate of 35°C/min. After combustion to CO_2, the stable carbon isotope ratios were determined on a Delta V isotope ratio mass spectrometer (Thermo Finnigan, Bremen, Germany).

Table S1 Overview of the characteristics of the groundwater used to prepare microcosms in 2009 and 2015

Parameter	Unit	2009	2015
Temperature	°C	12	11
pH		7.5	6.9
Conductivity	µS/cm	2080	2040
Oxygen	mg/l	0.3	1.1
Dissolved organic carbon	mg/l	17.0	32.0
Nitrite	mg/l	<0.03	<0.01
Nitrate	mg/l	<0.9	<0.05
Sulfate	mg/l	8.3	1.8
Iron (total)	mg/l	NA	21.0

NA means no iron (total) measured

Table S2 Average MtBE, TBA and benzene concentrations in groundwater per layer in µM

**Depth below surface level [m]**	**2009**			**2013**			**2015**		
	**MtBE**	**TBA**	**benzene**	**MtBE**	**TBA**	**benzene**	**MtBE**	**TBA**	**benzene**
5-10	534	127	63	14	41	32	9	37	27
10-15	6	-	13	4	7	66	1	7	18
15-20	26	24	10	5	23	132	0	16	49
20-25	2	-	0	1	20	5	0	28	0
25-30	5	-	1	-	-	-	0	1	1
**Average (5-25)**	**142**	**75**	**22**	**6**	**23**	**59**	**3**	**22**	**24**

Table S3 The amount of electrons released from MtBE and/or benzene, assuming complete oxidation to CO_2, and the amount of electrons used to reduce sulfate to sulfide, nitrate to nitrogen, iron(III) to iron(II) and chlorate to chloride

Condition	Consumed substrate* [μM]	Electrons released from MtBE and/or benzene [μeq]	Electrons used to reduce acceptor [μeq]
Nitrate addition [mol]			
- medium	ND	ND	ND
+ medium with 5% liquid inoculum	670 (B)	20095	23500
Chlorate addition [mol]			
- medium	49 (B)	1461	12342
+ medium	91 (B)	2741	11472
Iron addition [mol]			
- medium	77 (B) + 79 (M)	4654	180
+ medium	74 (B) + 510 (M)	17515	148
Sulfate addition [mol]			
- medium	158 (B) + 78 (M)	7061	11609
+ medium	115 (B) + 286 (M)	12012	14470

ND means no degradation up to 940 days

*Consumed substrates were benzene (B) and/or MtBE (M)

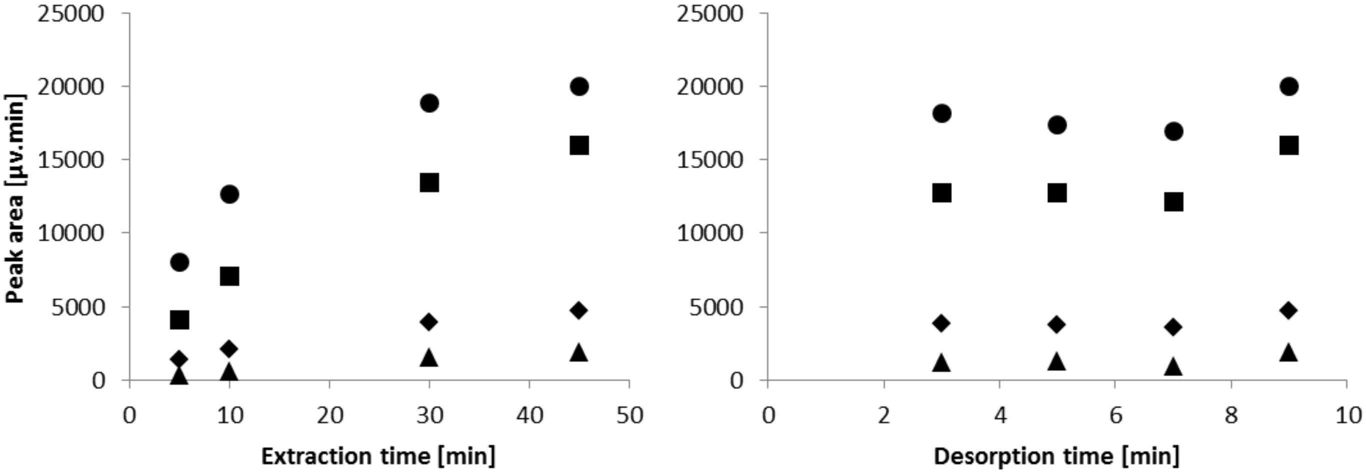

Figure S1. Determination of the optimal extraction and desorption time for the GC-FID SPME analysis for MtBE (diamonds), EtBE (squares), TBA (triangles) and benzene (circles)

References

David F, Sandra P (2007) Stir bar sorptive extraction for trace analysis J Chromatogr A 1152:54-69
